# Supplementary material for: Development, Optimisation and Validation of a Novel Multiplex Real-Time PCR Method for the Simultaneous Detection of Cryptosporidium spp., Giardia duodenalis and Dientamoeba fragilis
Source: Pathogens. 2022 Oct 31;11(11):1277. doi: 10.3390/pathogens11111277 (PMC9693193; doi:10.3390/pathogens11111277)
Supplement: Supplementary file 1 [file pathogens-11-01277-s001.zip › Table S1 pathogens-1997170.pdf]

pathogens MDPI

Supplementary Materials

Table S1. Diagnostic performance of commercially available multiplex real-time PCR assays for the simultaneous detection of *Cryptosporidium* spp., *Giardia duodenalis*, and *D. fragilis*.

Assay	Manufacturer	Pathogen species	Sensitivity (%)	Specificity (%)
AllPlex GI Panel	Seegene	*Cryptosporidium* spp.	1	0.99–1
		G. duodenalis	0.97–0.99	0.98–1
		D. fragilis	0.97–1	0.94
EasyScreen	Genetic Signatures	*Cryptosporidium* spp.	1	1
		G. duodenalis	0.92	1
		D. fragilis	0.95	1
FTD Stool Parasites	Fast Track	*Cryptosporidium* spp.	0.53–0.98	0.99–1
		G. duodenalis	0.90–1	0.93
		D. fragilis	1	0.98–1
NanoCHIP	Savyon Diagnostics	*Cryptosporidium* spp.	0.98–1	0.95–1
		G. duodenalis	0.98–1	0.95–1
		D. fragilis	1	1
RIDAGENE	R-Biopharm	*Cryptosporidium* spp.	0.86–0.92	1
		G. duodenalis	0.92	0.99
		D. fragilis	0.25–0.79	0.98
Tib MolBiol	Roche Diagnostics	*Cryptosporidium* spp.	0.86	0.99
		G. duodenalis	0.97	0.99
		D. fragilis	0.99	0.99
